# Supplementary material for: Beyond traditional stimuli: Validating AI-generated images for eliciting negative emotions in affect research
Source: PLoS One. 2026 Feb 10;21(2):e0342434. doi: 10.1371/journal.pone.0342434 (PMC12890096; doi:10.1371/journal.pone.0342434)
Supplement: S1 Table — Example prompts were used in the three AI platforms in early 2024. Newer AI-generative models will produce different outputs when using the above parameters and text prompts. (DOCX) [file pone.0342434.s001.docx]

**S1 Table. Example Prompts and Generated Outputs from Adobe Firefly, Stable Diffusion and Leonardo.ai**

| **AI Image Generator** | **Emotion** | **Original AI-generated Stimuli** | **Final rated stimuli** | **Prompt** | **Additional Parameters** |
| --- | --- | --- | --- | --- | --- |
| Adobe Firefly (v2.0) | Neutral | 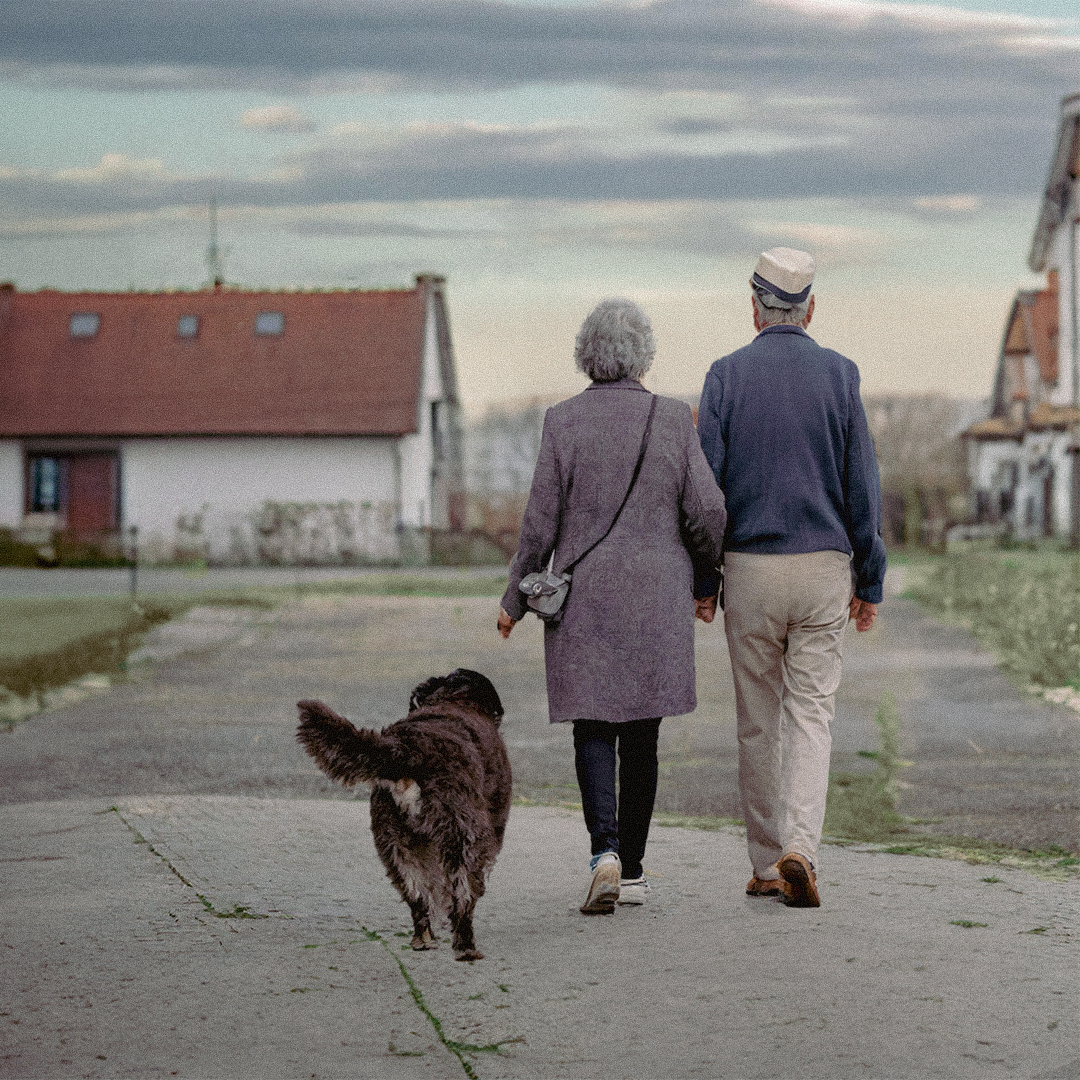 | 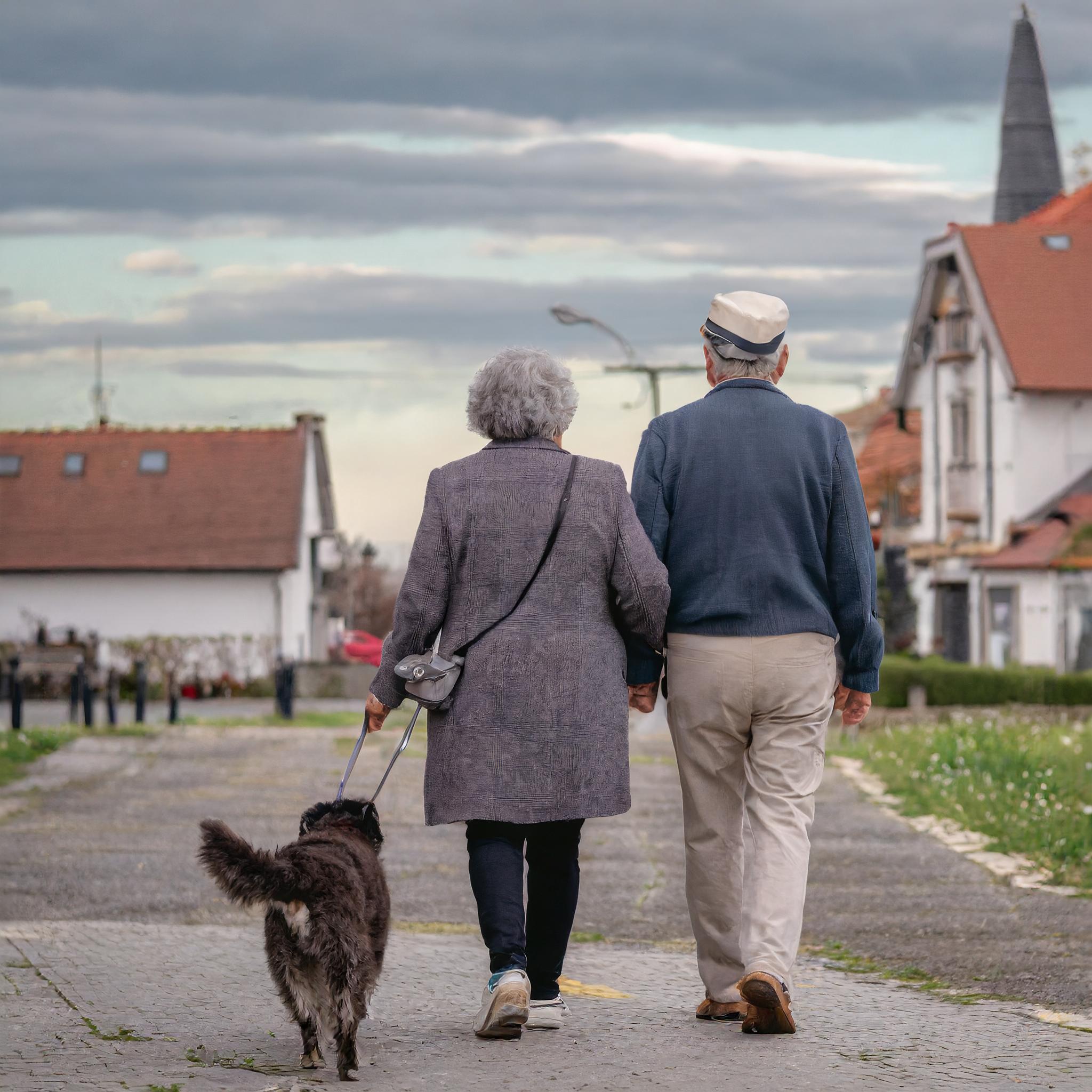 | “Back view of an elderly couple walking a dog, passing by a rural suburban town square; medium distanced shot” | None specified |
|  | Neutral | 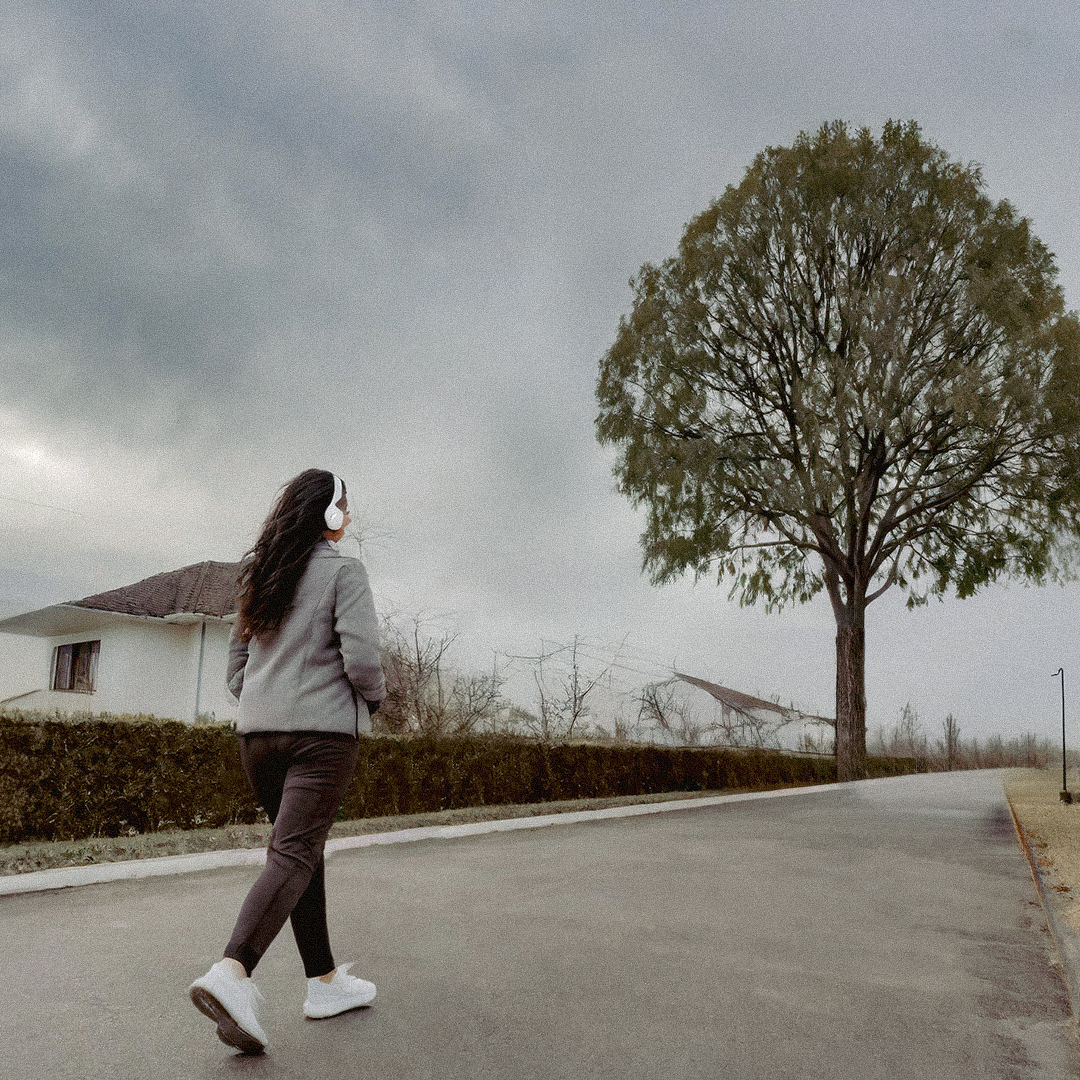 | 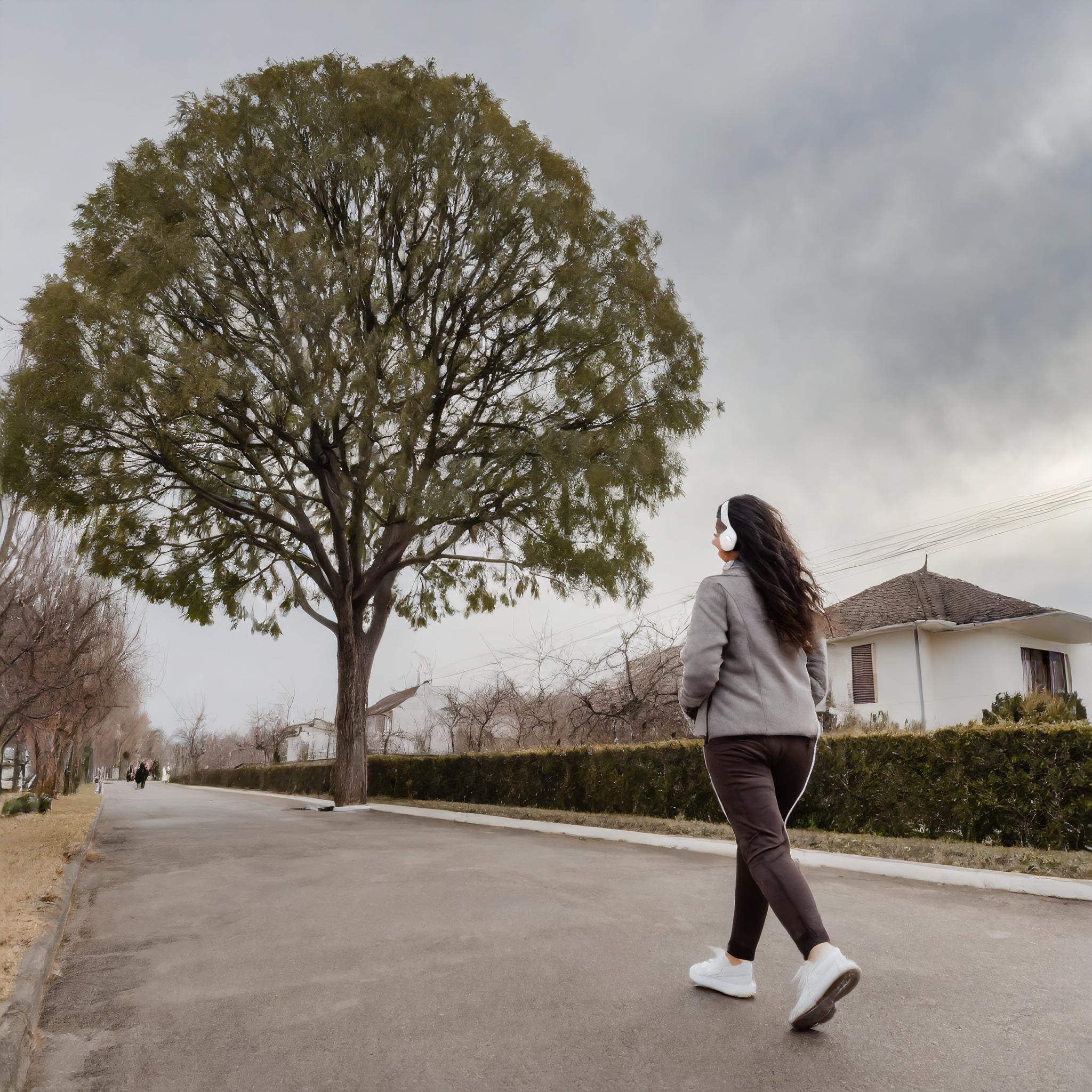 | “View of a person walking while listening to music on earphones on the suburb streets  with a grey sky” | None specified |
| Leonardo.ai | Negative | 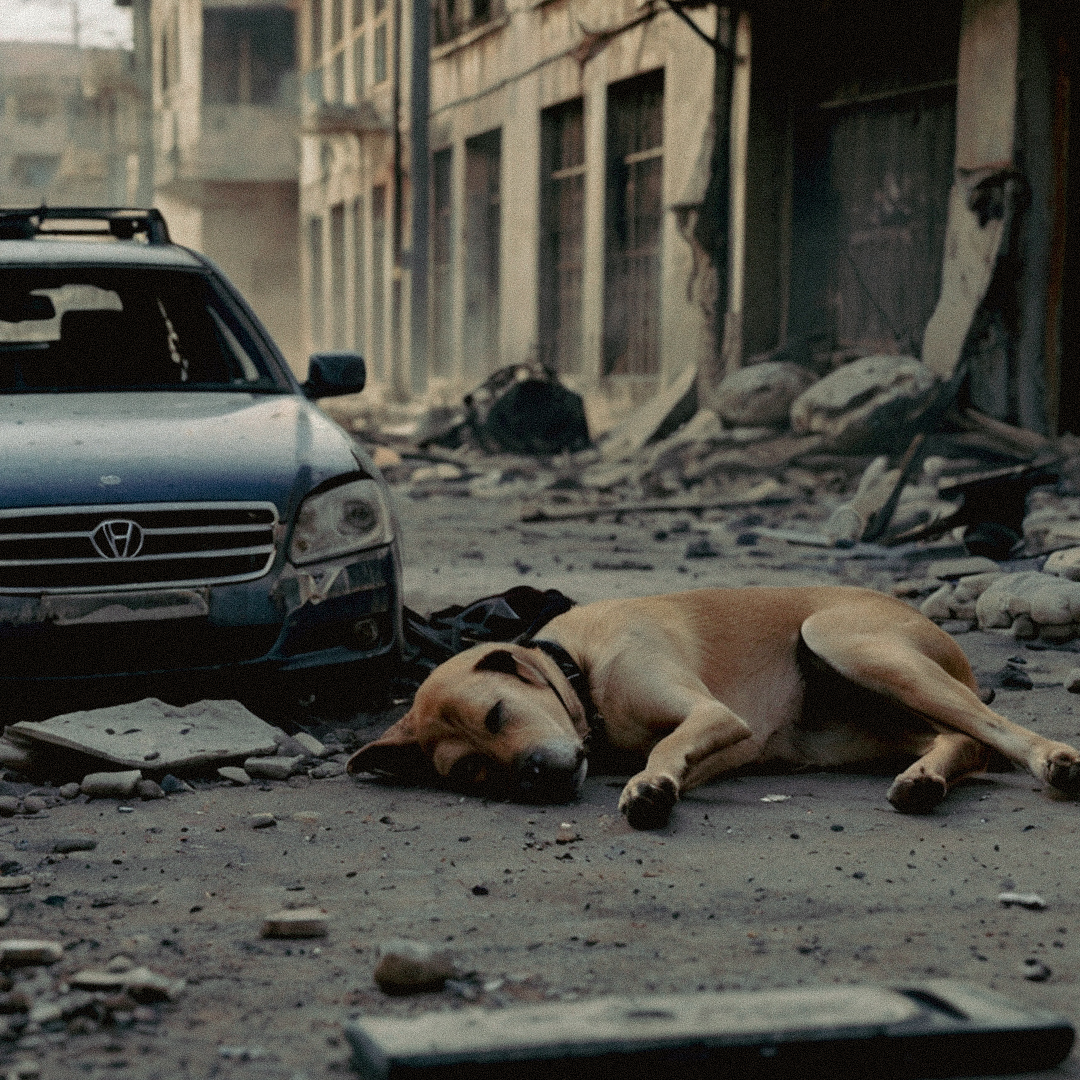 | 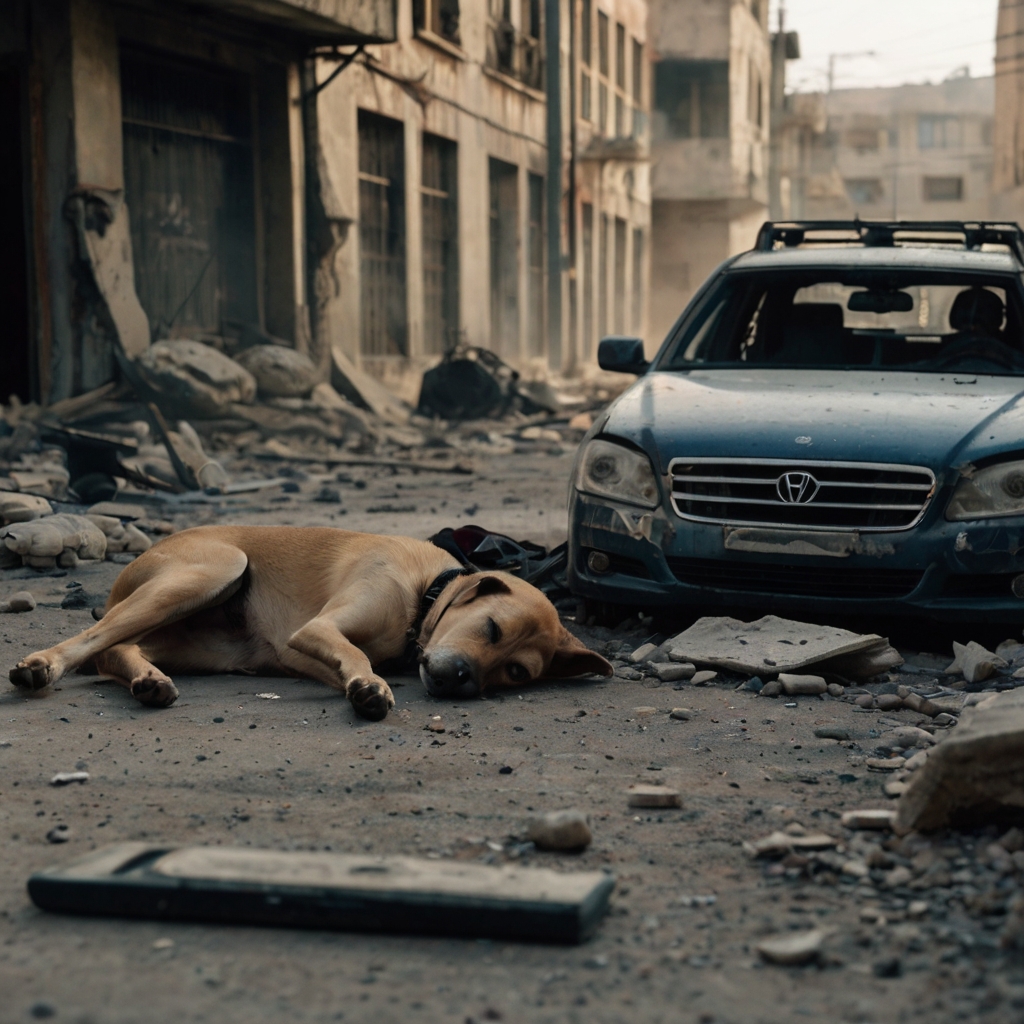 | “A car in a warzone; a dog is lying on the ground unconscious” | - Finetuned model: Leonardo Kino XL - Seed: 571804928 - Preset Style: None - Generation mode: Fast |
|  | Negative | 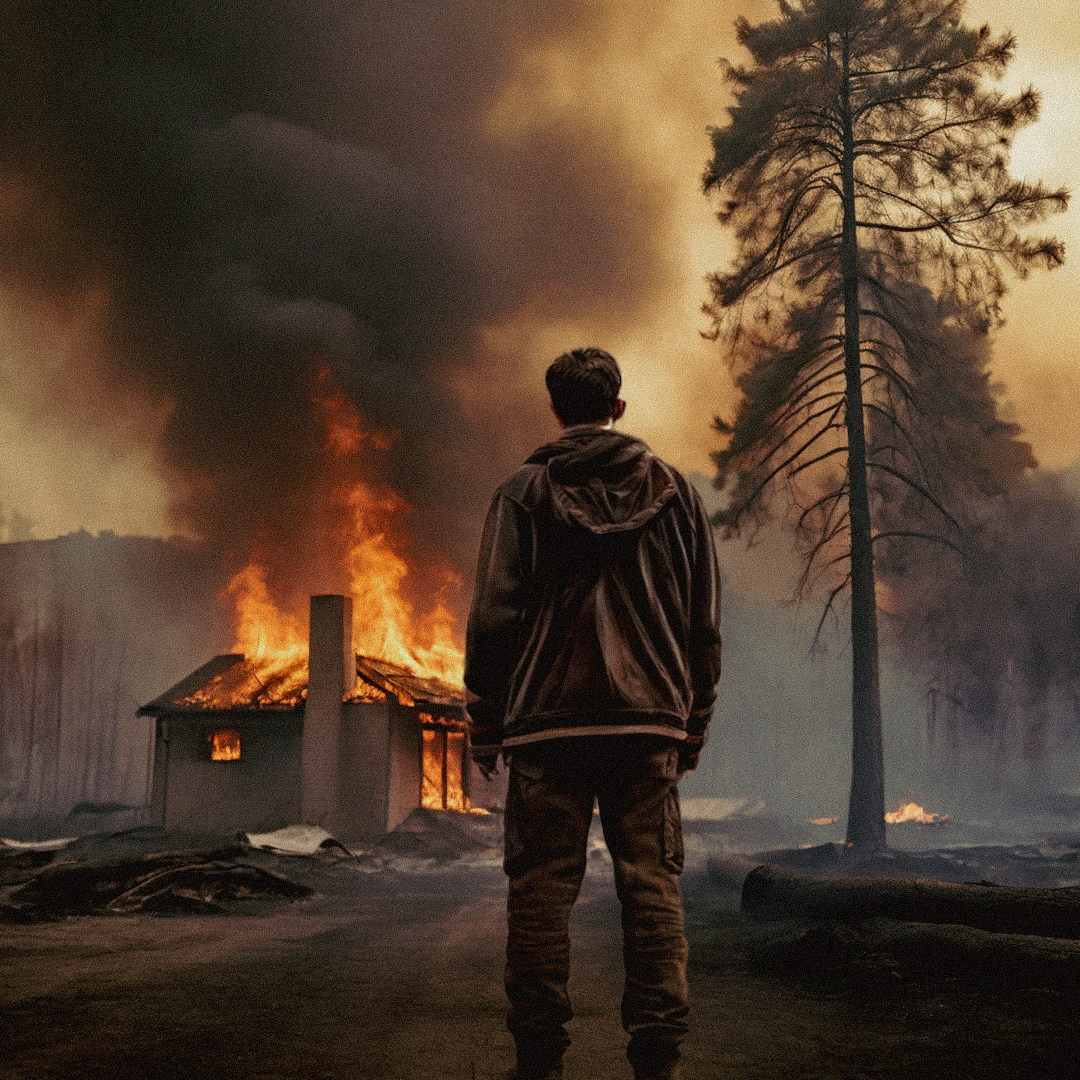 | 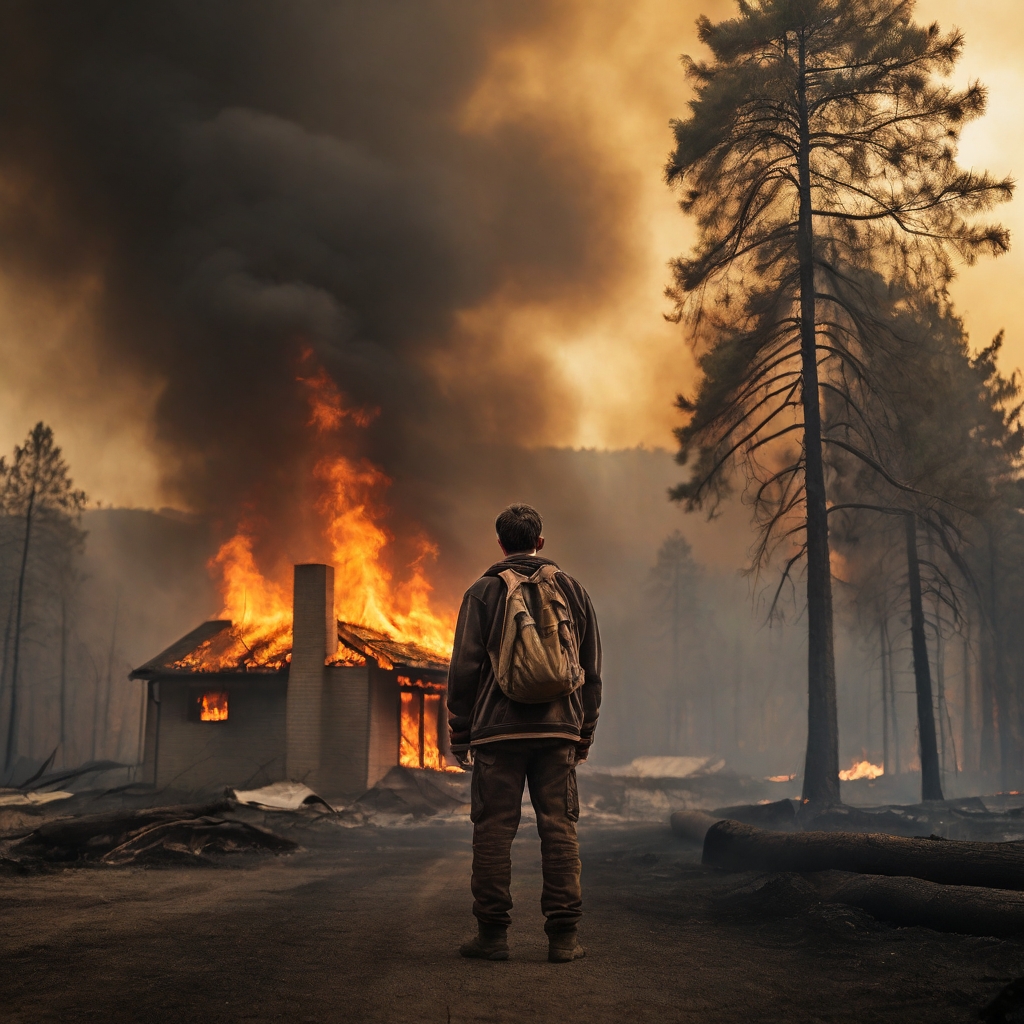 | “Back view of a man losing his home to a forest fire; sad and hopeless atmosphere; a tree nearby is on fire” | - Finetuned model: Leonardo Diffusion XL - Seed: 886756864 - Preset Style: Leonardo style - Generation mode: Fast |
| Stable Diffusion | Negative | 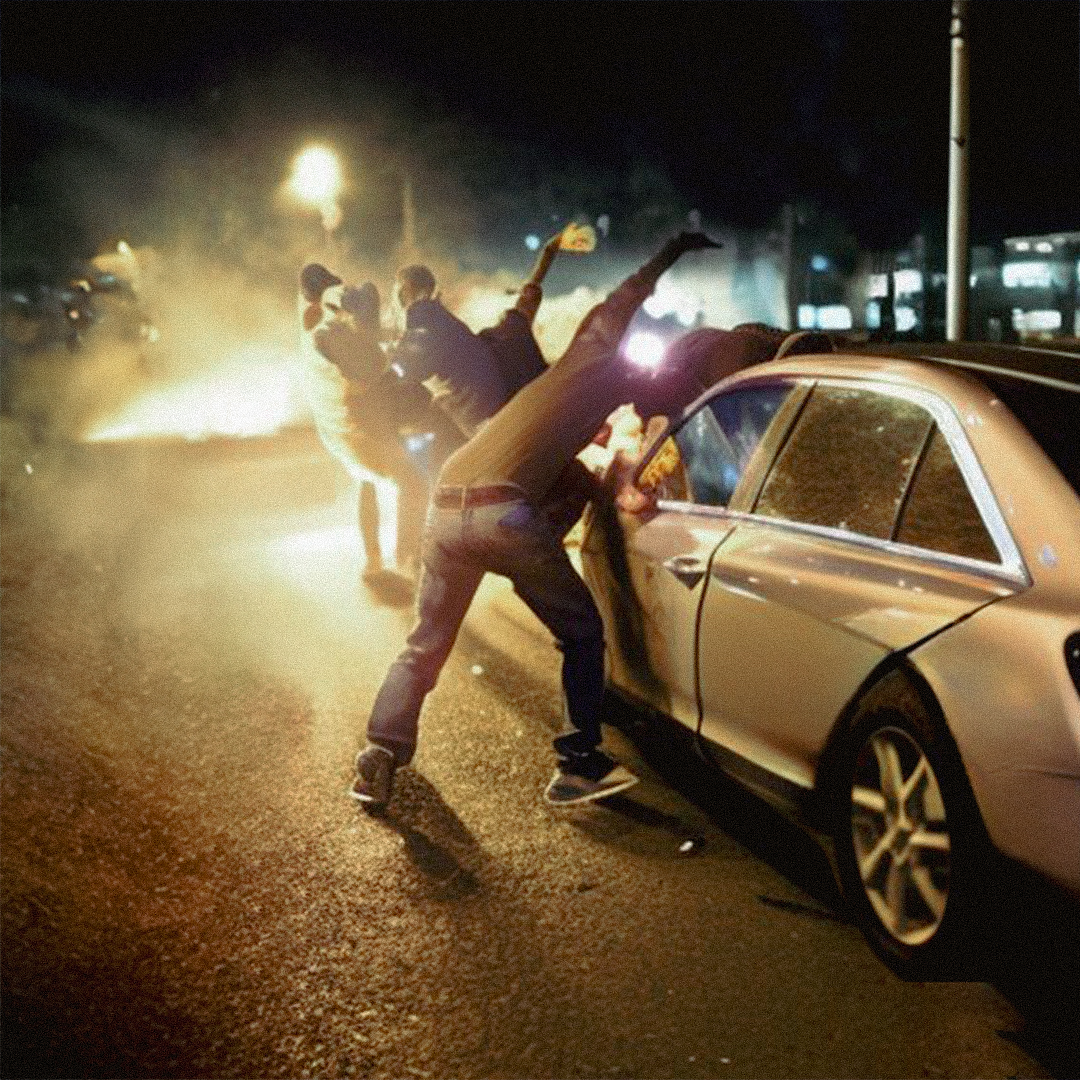 | 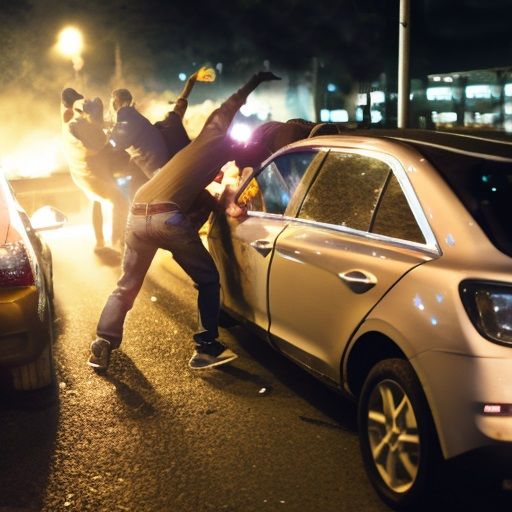 | “Photo of a group of people assaulting a man right next to a car during night time” | - Prompt strength: auto - Generation steps: 150 |
|  | Negative | 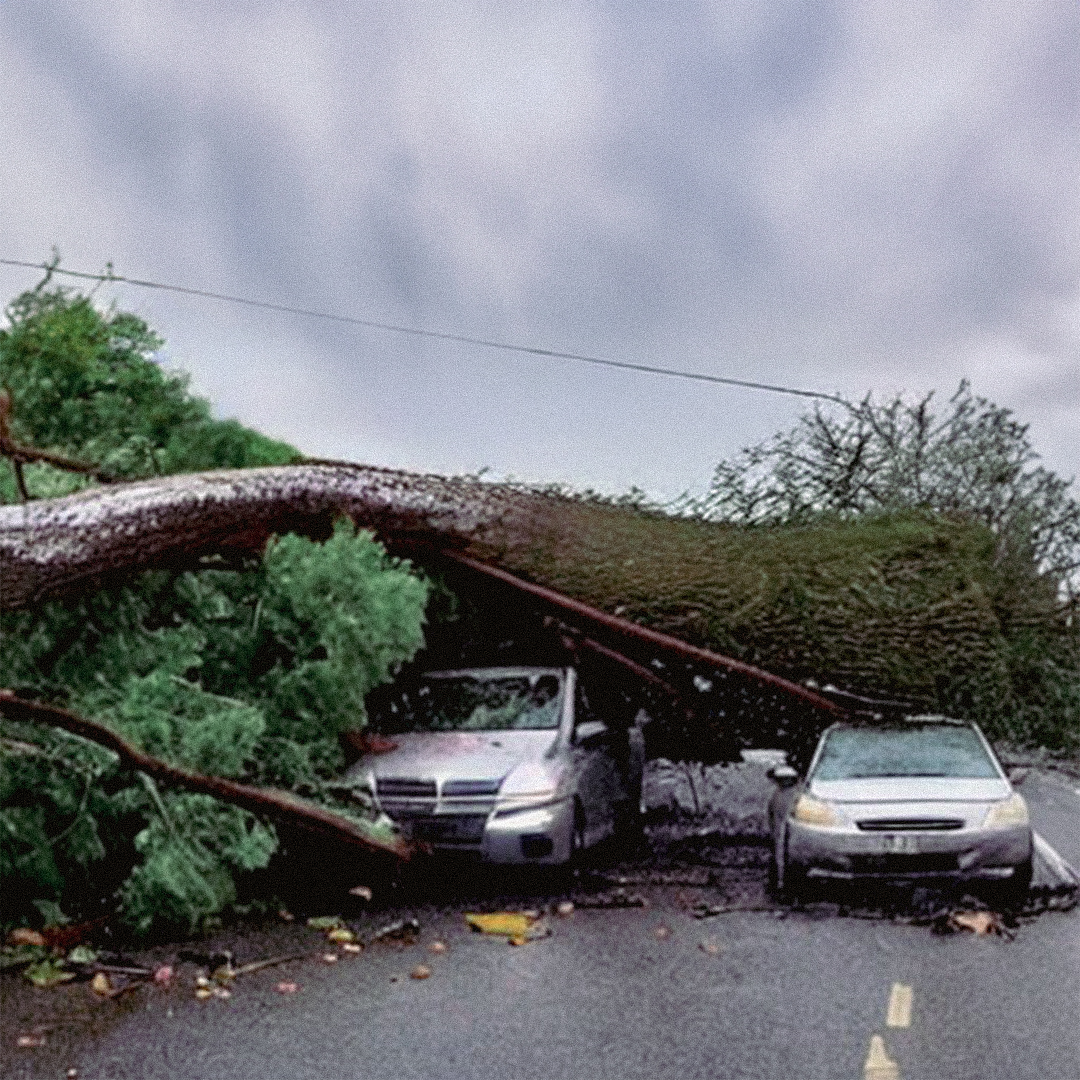 | 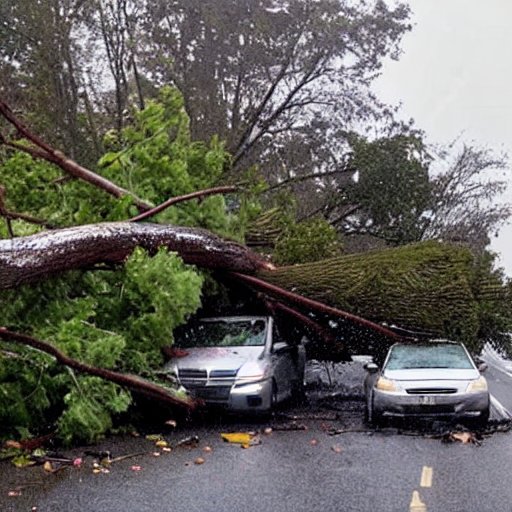 | “Photo of a tree falling onto a car on the road that damages the car” | - Prompt strength: auto - Generation steps: 150 |

*Note*. Example prompts were used in the three AI platforms in early 2024. Newer AI-generative models will produce different outputs when using the above parameters and text prompts.
