# Supplementary material for: Beyond traditional stimuli: Validating AI-generated images for eliciting negative emotions in affect research
Source: PLoS One. 2026 Feb 10;21(2):e0342434. doi: 10.1371/journal.pone.0342434 (PMC12890096; doi:10.1371/journal.pone.0342434)
Supplement: S4 Table — M and SD = mean and standard deviation, respectively. (DOCX) [file pone.0342434.s004.docx]

**S4 Table. Descriptive statistics for valence and arousal ratings for participants who noticed the AI-generated images (“Aware”) versus those who did not (“Unaware”) in Study 1 and Study 2**

| **Study 1** | **Aware (*n* = 11)** | | | | **Unaware (*n* = 54)** | | | |
| --- | --- | --- | --- | --- | --- | --- | --- | --- |
|  | **Negative** | | **Neutral** | | **Negative** | | **Neutral** | |
|  | ***M*** | ***SD*** | ***M*** | ***SD*** | ***M*** | ***SD*** | ***M*** | ***SD*** |
| Valence | 2.50 | 0.62 | 5.66 | 0.74 | 2.55 | 0.58 | 5.84 | 0.76 |
| Arousal | 4.87 | 0.81 | 3.30 | 0.73 | 5.20 | 0.61 | 3.89 | 0.65 |

| **Study 2** | **Group 1** | | | | | | | |
| --- | --- | --- | --- | --- | --- | --- | --- | --- |
|  | **Aware (*n* = 13)** | | | | **Unaware (*n* = 30)** | | | |
|  | **Negative** | | **Neutral** | | **Negative** | | **Neutral** | |
|  | ***M*** | ***SD*** | ***M*** | ***SD*** | ***M*** | ***SD*** | ***M*** | ***SD*** |
| Valence | 3.04 | 0.65 | 5.72 | 0.59 | 2.60 | 0.62 | 5.70 | 0.72 |
| Arousal | 4.42 | 0.77 | 3.11 | 0.72 | 5.38 | 0.78 | 3.87 | 0.61 |
|  | **Group 2** | | | | | | | |
|  | **Aware (*n* = 6)** | | | | **Unaware (*n* = 32)** | | | |
|  | **Negative** | | **Neutral** | | **Negative** | | **Neutral** | |
|  | ***M*** | ***SD*** | ***M*** | ***SD*** | ***M*** | ***SD*** | ***M*** | ***SD*** |
| Valence | 2.61 | 0.66 | 5.77 | 0.81 | 2.66 | 0.57 | 5.67 | 0.82 |
| Arousal | 5.00 | 0.87 | 2.96 | 1.06 | 5.29 | 0.88 | 3.20 | 0.75 |

*M* and *SD* = mean and standard deviation, respectively.
