# Supplementary material for: Beyond traditional stimuli: Validating AI-generated images for eliciting negative emotions in affect research
Source: PLoS One. 2026 Feb 10;21(2):e0342434. doi: 10.1371/journal.pone.0342434 (PMC12890096; doi:10.1371/journal.pone.0342434)
Supplement: Attachment — Submitted filename: Response to Reviewers_sa_v2.docx [file pone.0342434.s006.docx]

We greatly appreciate the reviewer’s comments on the manuscript. Below, we have organized the comments that require revision and our point-by-point response to each comment.

**Reviewer #1:**

1. The Manuscript needs careful English proofreading because there are some shortcomings. For instance, the article “the” is sometimes missing in front of nouns, the message in some paragraphs is not clear enough. It looks like the first part was written by one author with a greater command of the English language, and the rest of the paper was written by someone else. The numerous grammar errors made this a difficult paper to read. It was strange to see the authors refer to tables that were not submitted. I was unable to find any supplementary material to the submission, so I think this was truly omitted by the authors. Please read the manuscript carefully.

- *Table 1-5 are enclosed within the main text of the PDF file, which can be viewed using Acrobat Reader. Table S1 and S2 are provided as supporting information and can be accessed by clicking the download links at the end of the PDF file (pages 53 and 54). After accepting the download request, the supporting information in Word document format should be automatically downloaded to the default download folder of the local computer. While other reviewers have not reported a similar issue with accessing the supporting information, we suspect that the problem might be due to different PDF readers. Therefore, we recommend using Acrobat Reader for viewing the PDF file. Additionally, we have carefully proofread the manuscript and made necessary changes to enhance its readability.*

2. The abstract must contain the main purpose of the paper, the research method used in the research and the main contributions.

- *The abstract has been revised accordingly to clarify the aims and scope of this paper.*

3. It would be very useful to add in the "Introduction" section the purpose, objectives and hypothesis of the research. I consider that a weak point of the paper is that the authors did not show the novelty of the paper compared to other works. That is why, I consider that the introduction should specify the novelty of the paper compared to other papers published in this area.

- *We have expanded on our literature review on current uses of AI in stimuli development by including more recent studies of AI-generation of facial stimuli (lines 84-89). We have also clarified in the Research Gap and Present Study section that the novelty of this study is to systematically examine the affective dimensions of AI-generated naturalistic scene stimuli (lines 111-119), with the aim of demonstrating that the normative ratings are replicable across independent samples (lines 130-139)*

4. The Literature Review cannot be missing from the paper.

- *Although there is no dedicated subheading for the literature review, it is incorporated into the introduction section.*

5. The research is well based on science and the results are in agreement with the theoretical part.

- *We appreciate the positive comments from the reviewer.*

6. I believe that the authors should also include other indicators from Descriptive Statistics.

- *Descriptive statistics are presented in the main text and Table 2 and 4.*

7. I think that the literature needs to be improved with other recent works, refers to the companies listed on the economic growth. That is why I recommend the authors to refer to other recent works indexed in Web of Science, Scopus, Emerald and Cambrige Journals. We suggest that the authors cite papers published in Web of Science Journals such as:
   a. <https://doi.org/10.3390/cancers15030843>
   b. <https://doi.org/10.3390/s23052398>
   c. <https://doi.org/10.3390/math11102305>
   d. <https://doi.org/10.3390/fractalfract8100604>

In conclusion, the article should be improve. It should also be enhanced with a review of the literature adequate to the subject and a broader interpretation and commentary of the research results.

- *We appreciate the reviewer’s suggestions and carefully examined the four recommended papers from a pioneering research group that applies advanced mathematical and computational models to complex classification and detection problems across diverse scientific domains. While our study focuses on using existing AI tools to generate negative and neutral images for affective research, we found limited conceptual or methodological overlap with these works. Accordingly, we did not include citations to these papers in the manuscript.*

**Reviewer #2:**

1. First, it is not clear why the authors have two sub-studies. Study 1 and Study 2 overlap, and it seems obvious that Study 2 is more rigorous than Study 1. The authors stated that the participants in Study 1 saw the stimulus images before Study 1 as part of another study. This is a very significant limitation, which I think the authors cannot get away with. As mentioned in the manuscript as well, habituation effects likely happened. Since the authors did not explain what kind of study the previous study was and in what context the images were displayed, it is difficult to guess how seriously habituation effects occurred there. But, one clear thing is that the previous experience must have harmed the quality of Study 1 to a certain extent. My best guess about why the authors included Study 1 is probably that the consistent findings across the two sub-studies can demonstrate the reliability of their findings. However, I would say that is not the case here, due to the critical limitation of Study 1. Therefore, I suggest the authors retain only Study 2.

- *We thank the reviewer for this comment and have revised the manuscript (lines 152-165) to clarify the distinct and complementary roles of Study 1 and Study 2.*

*A primary objective of this paper is to demonstrate the feasibility of using generative AI to create stimuli for affective research. Study 1 was designed to reflect a common practice in the field, where affective ratings are collected from participants after the main experiment. The findings confirmed that the AI-generated negative and neutral stimuli elicited different valence and arousal ratings. However, this design introduced a potential confound of habituation, as the ratings were collected upon the participants' second exposure to the images.*

*To address this limitation and establish the reliability of our findings, we conducted Study 2. This study provided a methodologically clean validation by collecting ratings upon the first exposure to the stimuli, thereby removing any potential influence of habituation.*

*By presenting both studies, we demonstrate that the AI-generated images exhibit consistent affective characteristics, regardless of whether ratings are collected during the first or second exposure. This dual-study approach provides a robust validation of the stimuli and strengthens the basis for our recommendation in the discussion: researchers who generate their own stimuli should collect normative ratings after the main experiment if collecting them from a separate, naive sample is not feasible.*

2. Another key limitation of this study is that the study did not test positive images. Although the authors clearly admit this as their limitation in the Discussion section, I do not think this is a simple limitation that they can admit and move on. In fact, not examining positive images makes this study’s findings look half-baked. The true utility of AI-generated images as tools for valence/emotion research can be only confirmed after testing positive images (and positive valences). If there was an unavoidable reason behind this critical exclusion, the authors should clarify it in the manuscript. Without that, the current version leaves some doubt about the completeness of the study.

- *We have now included a detailed explanation of focusing solely on evaluating AI-generated negative and neutral images (lines 140-151) from the theoretical, clinical and practical consideration in the introduction and discussed the potential limitation in the discussion section. We have also revised our study title to clarify that our evaluation of valence and arousal patterns is limited to the negative-neutral spectrum. As a first study, we prioritized evaluating negative versus neutral images due to the theoretical and clinical importance of negative affect processing. Including both negative and positive images in the same study would inevitably increase the total number of images participants needed to view and rate (for around 50% more images), potentially compromising the reliability of the ratings due to fatigue and habituation. After weighing the pros and cons of different designs, we decided to focus on the AI-generated negative image in this study and acknowledged in the limitations section that this methodological constraint limited the generalizability of our results (lines 569-574).*

**Reviewer #3:**

1. Despite these strengths, the study has several serious limitations that must be addressed before it can be considered for publication. Most notably, the study excludes positive stimuli entirely, undermining the central claim that AI-generated images can replicate the full range of affective responses typically captured in standardized image sets. Without positive images, the U-shaped valence–arousal distribution is only partially validated, which significantly limits the generalizability of the findings. The authors must clearly justify this omission and discuss its implications. … While the authors briefly acknowledge the absence of positive images and the choice to include AI-distorted outputs, these decisions require more detailed justification and discussion. Specifically, the complete omission of positive stimuli significantly limits the ability to validate the full valence–arousal spectrum and undermines the claim that AI-generated images replicate the distributional patterns observed in standardized databases like IAPS. The rationale for this exclusion is not sufficiently developed.

- *We thank the reviewer for highlighting this important issue. We agree that excluding positive images limits our ability to characterize the full valence–arousal distribution observed in standardized databases. In response, we have (i) added a more detailed rationale in the Introduction (lines 140-151) for focusing on AI-generated negative and neutral images, drawing on theoretical, clinical, and practical considerations; and (ii) explicitly discussed this limitation in the Discussion (lines 569-574). We have also revised the title of the manuscript to clarify that our evaluation of valence–arousal patterns is restricted to the negative–neutral spectrum.*

*As an initial study, we prioritized negative versus neutral affect given the theoretical and clinical salience of negative affect processing. Including positive images in the same protocol would have increased the number of stimuli by approximately 50%, raising risks of participant fatigue and habituation and, in turn, potentially reducing rating reliability. After weighing these trade-offs, we elected to focus on AI-generated negative (and neutral) images in this study and to acknowledge the resulting limits on generalizability in the Limitations section (lines 569-572). We view a balanced inclusion of positive stimuli as a key direction for subsequent work designed specifically to test the full valence–arousal space.*

2. Second, no data were collected on whether participants recognized the images as AI-generated. This omission is problematic, as prior work suggests that perceived

artificiality can modulate emotional responses. Furthermore, the authors report that images with visual distortions or unrealistic elements were intentionally retained. While this decision may reflect practical constraints, it raises concerns about ecological validity and the applicability of findings to real-world emotional processing. Additionally, although the authors note that realism was not a primary goal, they do not assess whether participants were aware that the images were AI-generated or whether such awareness might have influenced their emotional ratings. Given growing evidence that perceived authenticity can modulate affective responses, this represents a potential confound that should be directly addressed. Both points—the exclusion of positive stimuli and the lack of participant awareness checks—require clearer theoretical and methodological justification.

- *We did not formally obtain data on whether participants recognized the images as AI-generated. However, informally at the end of each study, we asked participants about their initial perception of the stimuli, but this data is not standardized enough to be included in our analysis. Whether recognizing the origin of the images will influence emotional responses is not clear cut. Some recent studies (Zhou & Kawabata, 2023; Bilucaglia et al., 2025) have indicated implicit effects of participants’ awareness, but this effect does not extend to explicit subjective ratings of valence and arousal. This discussion is now included in the manuscript in the limitations section (see lines 574-586)*

*The main purpose of the AI-generated images was for emotional elicitation in the context of typical experimental tasks in affective research. Considering that the presentation time of the images in these tasks is fairly short (e.g., few seconds), we did not try to modify the generated images to ensure that they were perceived as ‘real’. This has been explained in the method section (lines 231-238) and also in the discussion (lines 559-568)*

3. The paper provides only a few examples of the generated images and their corresponding prompts, which raises concerns about transparency and reproducibility. Given that the study’s main contribution lies in the use of generative AI for flexible stimulus creation, it is essential to disclose a representative and sufficiently detailed sample of prompts, along with generated outputs. Without this information, it is difficult to assess the thematic accuracy, variability, or potential biases in the generation process. Most critically from a methodological standpoint, the authors do not address the inherent stochasticity of text-to-image generation models. Most generative AI systems sample from a probability distribution, meaning that identical prompts can yield different images across runs—especially on platforms like Adobe Firefly and DALL·E, which do not allow for control over random seed values or generation parameters. This severely compromises the reproducibility of the stimuli. The authors’ proposal that AI-generated images could replace standardized image databases is therefore premature unless procedures are put in place to ensure deterministic, shareable outputs. At a minimum, the authors should document the AI model configuration and any image metadata. If the same prompt was used in both Study 1 and Study 2, it would be valuable to examine and report whether the emotional ratings of the resulting images differed—highlighting the variability introduced by prompt reuse without seed control.

- *While our findings showed that AI-generated images can be valid stimuli for affective research in our studies, we did not intend to replace standardized image databases with these AI-generated images. Instead, we aim to demonstrate the feasibility of generative AI as a powerful complementary tool that allows researchers to flexibly generate their own set of affective stimuli tailored to specific experiment requirements. We also show how to validate these AI-generated stimuli, either by the same group of participants after the main experiment (Study 1) or by a separate group of participants who were not exposed to the stimuli before the rating (Study 2). Although we never intend to suggest researchers replicate the same set of affective stimuli using the prompts we used, we’ve discussed the challenges of reproducibility in the manuscript (lines 553-558) and provided additional text prompts, generated outputs, and modified images from our dataset. These prompts represent those used across Adobe Firefly, Leonardo.ai, and Stable Diffusion platforms (see S1 Table, supplementary). While these prompts will not recreate the exact image, they still offer value in helping researchers develop stimuli with similar themes. We also explicitly state that readers can request access to our negative and neutral AI stimuli (see lines 164-165).*
